# Exercise testing in athletes: an adjunct to diagnose arrhythmogenic cardiomyopathy?

**DOI:** 10.3389/fcvm.2026.1900090

**Published:** 2026-07-20

**Authors:** Simon Wernhart, Martin Halle, Mark J. Haykowsky, Satyam Sarma

**Affiliations:** 1 Department for Preventive Sports Medicine and Sports Cardiology, TUM School of Medicine and Health, TUM University Hospital, Technical University of Munich (TUM), Munich, Germany; 2 DZHK (German Centre for Cardiovascular Research), Partner Site Munich Heart Alliance, Munich, Germany; 3 Integrated Cardiovascular and Exercise Physiology and Rehabilitation (iCARE) Laboratory, College of Health Sciences, University of Alberta, Edmonton, AB, Canada; 4 Department of Cardiology, Hochgebirgsklinik, Davos, Switzerland; 5 Department of Internal Medicine, University of Texas Southwestern Medical Center, Dallas, TX, United States; 6 Institute for Exercise and Environmental Medicine, Texas Health Presbyterian Hospital Dallas, Dallas, TX, United States

**Keywords:** arrhythmogenic cardiomyopathy, cardiac output, exercise phenotyping, exercise testing, oxygen consumption

## Abstract

Differentiating physiological adaptation to exercise from cardiomyopathic hearts can be challenging. Specifically, evidence of how to deal with athletes under suspicion of arrhythmogenic cardiomyopathy (ACM) but not fulfilling diagnostic criteria is scarce and necessitates a better understanding of exercise pathophysiology and prospective trials. Upon available literature, we discuss and suggest research on combined stress echocardiography and cardiopulmonary exercise testing (CPET echocardiography) to demonstrate mismatch between cardiac output and metabolic demand. Integrating CPET echocardiography in suspected ACM cases may be feasible and could help to better differentiate physiological adaptation to exercise from cardiomyopathic hearts and may become a valuable adjunct for risk stratification and diagnostic frameworks. Future studies should implement combined CPET and stress echocardiography to differentiate physiological adaptations to exercise from cardiomyopathies in athletes with suspected ACM not fulfilling diagnostic criteria.

## 1 Introduction

Arrhythmogenic cardiomyopathy (ACM) is a hereditary disease with a primarily autosomal dominant trait with variable penetrance and is a major reason for sudden cardiac death during sport (1). The Task Force (2) and modified Padua criteria to diagnose ACM include morphological-structural abnormalities including tissue characterization, functional, electrocardiographic, familial history, identification of pathogenic genetic variants, as well as ventricular arrhythmia with a critical appraisal of the family history of the index patient (3). Despite broader availability of genetic testing and advanced cardiac imaging, ACM remains a challenge to diagnose and risk stratify.

Diagnosis of ACM is particularly challenging in athletes and has major implications for exercise recommendations. Intense (endurance) exercise has been proposed to accelerate disease progression and risk of malignant arrhythmias in ACM, which is largely based on retrospective data from patients with plakophilin-2, PKP-2, variants (4). Although the overall risk of malignant arrhythmia is high in ACM, a precise and causal dose-event relationship between training volume (intensity and duration as well as the relationship between exercise stimuli and resting periods) and the risk of arrhythmia has not been established (4). This is acknowledged in a current position paper and critical review of exercise recommendations, stating that counselling athletes to refrain from competitive sports in non-PKP-2 ACM lacks prospective scientific evidence (4, 5). Importantly, the risk of arrhythmia seems to be dependent on both pheno- and genotype of the disease (4), illustrating the necessity for careful risk stratification (including geno- and phenotyping) and refinement of diagnostic criteria in athletes willing to continue exercise at a competitive level.

## 2 Current diagnostic challenges and potential for future research

Current recommendations to diagnose ACM (3) rely on resting measurements leaving diagnostic uncertainties in some highly trained athletes. Nearly one-third of elite athletes have shown right ventricular dilatation compatible with minor criteria and four percent meet major Task Force criteria, illustrating the overlap between the athletic and cardiomyopathic heart (4). Current position papers acknowledge the overlap between athletic and cardiomyopathic hearts (3, 4) which may make exercise stress testing a valuable adjunct to current diagnostic criteria for ACM.

**Figure 1:**
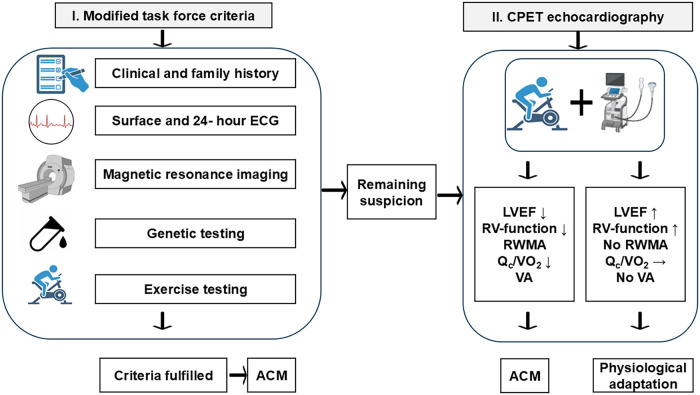
Proposed diagnostic algorithm for athletes with suspected arrhythmogenic cardiomyopathy. Clinical and family history, surface and 24- hour Holter electrocardiogram (ECG), magnetic resonance imaging, exercise and genetic testing represent standard baseline investigations. If diagnostic criteria for arrhythmogenic cardiomyopathy (ACM) are met, no further testing is required. If diagnostic criteria are not met and clinical suspicion remains, we recommend combined cardiopulmonary exercise testing and stress echocardiography (CPET echocardiography). ACM may be considered in case of a lack of increase or a decline of left ventricular ejection fraction (LVEF), regional wall motion abnormalities (RWMA), ventricular arrhythmias (VA), or reduced right ventricular (RV) function. A reduced cardiac output (Q_c_) to oxygen consumption (VO_2_) slope could suggest ACM rather than a physiological adaptation of the heart. In the presence of an increase in contractility (LVEF, RV-function) and absence of RWMA, physiological adaptation should be suspected. Adapted from “Proposed Diagnostic Algorithm for Athletes With Borderline Arrhythmogenic Cardiomyopathy” by Simon Wernhart, Martin Halle, Mark J. Haykowsky and Satyam Sarma, licensed under CC BY 4.0.

Detection of regional wall motion abnormalities on imaging or inadequate increase of ejection fraction during exercise may raise suspicion for underlying pathology and should not be found in the athletic heart (3). As resting ejection fraction can be physiologically reduced in well-trained athletes in a resting state, exercise testing has a pivotal role to demonstrate adequate stroke volume and heart rate reserve and normal matching of cardiac output and metabolic demand (Figure 1), which cannot be demonstrated by cardiopulmonary exercise testing (CPET) and stress echocardiography on their own.

## 3 Discussion

### 3.1 Physiological rationale

The relationship between cardiac output (Q_c_) and oxygen consumption (VO_2_) is independent of sex and age and remains unchanged across diseases (6). Q_c_ increases by 5–6 L/min for every 1 L/min increase in VO_2_ resulting in a physiological Q_c_/VO_2_ slope of 5–6 (6). Thus, a slope <5 may indicate central, while slopes >6 may illustrate peripheral limitations. In the absence of acute or chronic inflammation, which may impair peripheral oxygen extraction, athletes are not expected to show any signs of peripheral limitation. Athletes fulfilling borderline criteria for ACM may benefit from additional stress testing to demonstrate a mismatch between Q_c_ and VO_2_ during exercise. A slope <5 may herald underlying central limitations, which may be caused by subclinical ACM. However, it needs to be considered that in the context of high endurance training, stroke volume dynamics and heart rate reserve may exhibit specific adaptations at near-maximal intensities as highly trained endurance athletes may not demonstrate plateauing of stroke volume increase as usually seen in normal individuals (7). Thus, the linearity of the Q_c_/VO_2_ slope may not be maintained across all training volumes. High performing elite athletes might present borderline values that could mimic central limitation without underlying cardiomyopathy. Thus, a Q_c_/VO_2_ slope cut-off <5 to demonstrate cardiomyopathy-induced central limitations is hypothesis-generating in athletes and requires further exploration. Clearly, further studies are warranted in a cohort of athletes to validate Q_c_/VO_2_ slope as an additional tool of CPET echocardiography (simultaneous CPET and stress echocardiography).

According to Fick`s law, Q_c_ is proportional to VO_2_ and indirectly proportional to arteriovenous oxygen difference (avDO_2_): Q_c_ = VO_2_/avDO_2._ avDO_2_ is approximately 5 mL/100 mL blood at rest, while it increases to 15–20 mL/100 mL blood during aerobic exercise in athletes or patients (8–10). Thus, using CPET echo, avDO_2_ can be calculated from Q_c_ and VO_2_ at each stage of exercise. Although not measured directly (invasively), this is an extended sports cardiological approach to assess athletes during exercise compared to separated CPET or stress echo analysis. Measuring avDO_2_ directly, would require gaining both arterial and venous blood gases of the working muscles to accurately determine oxygen saturation and haemoglobin concentration; this is usually not feasible in routine investigation of athletes. Therefore, calculation of avDO_2_ from Q_c_ and VO_2_ is a feasible approach enabled by CPET echocardiography. While current sports cardiological guidelines and position statements recommend separate CPET and echocardiography, combined CPET echocardiography is not explicitly mentioned (4, 11).

### 3.2 Proposed diagnostic algorithm

Although the gold standard of assessment of Q_c_ during exercise is exercise right heart catheterization or exercise magnetic resonance imaging, these tools are expensive and not broadly available in sports cardiological facilities. Clinically applicable analysis of Q_c_ and VO_2_ could be implemented using combined stress echocardiography and CPET. As image quality in athletes is often satisfactory, stroke volume reserve can usually be obtained during semi-upright CPET (additionally, 3D imaging can be helpful). We have recently published a case report of an athlete not entirely fulfilling diagnostic criteria for ACM showing reduced central reserve during CPET echocardiography, which prompted us to be more restrictive on exercise recommendations after other potential reasons for central limitations, such as coronary artery disease, were ruled out (12). Cardiac reserve assessment during upright or semi-upright exercise may be preferred as it most closely matches the metabolic stress and position in which peak VO_2_ is assessed compared to standard tilted stress echocardiography. To minimize inter-operator variability and improve reproducibility of Q_c_-calculations, standardized protocols could be useful: For instance, pre-defined assessment of stroke volume and oxygen uptake at the first, second ventilatory threshold, peak performance, and a respiratory exchange ratio of 1.0. The value of non-invasive assessment of exercise physiology with CPET echocardiography, including lung ultrasound to screen for B-lines and exercise-induced pulmonary congestion supporting central limitation during exercise, has been acknowledged in patients with unexplained dyspnoea to phenotype heart failure patients (13–17). This approach could also be useful in athletes not entirely fulfilling diagnostic criteria for arrhythmogenic, but also in other cardiomyopathies, such as dilated (18) and hypertrophic (19, 20) cardiomyopathy, which may also display a mismatch between peripheral oxygen delivery and cardiac output. Whether Q_c_/VO_2_ < 5 unmasks hidden cardiomyopathy in athletes is speculative now and needs to be assessed in a prospective setting. We suggest the following protocol for a standardized procedure to study athletes with suspicion of underlying cardiomyopathy: (1) Resting measurements will be made to gain baseline CPET and echo values. We use a ramp protocol with an increment aiming at an exercise duration of eight to twelve minutes (21). However, workload will be halted and not further increased at specific moments to achieve adequate measurements at the first ventilatory threshold, RER = 1,0, second ventilatory threshold, and submaximal exercise (defined by a value of 18 on the BORG scale). Q_c_/VO_2_ slope will be calculated by linear regression analysis from all measured values.

We suggest implementing combined CPET echocardiography into the diagnostic workup of athletes with suspicion of ACM but not entirely fulfilling diagnostic criteria for ACM according to Task Force and modified Padua criteria. We have recently suggested a potential clinical workflow for borderline ACM (12), which has been adapted for our clinical routine and is also used for athletes under suspicion of other cardiomyopathies (Figure 1). Q_c_ and VO_2_ should be assessed at predefined steps of incremental exercise testing (such as the first and second ventilatory threshold) yielding enough measurements to calculate a proper slope (22). Physiological adaptation to exercise in a healthy athletic heart may yield a slope between 5 and 6, while Q_c_/VO_2_ < 5 may indicate central limitations and a restrictive phenotype, which may indicate subclinical (left, right or biventricular) ACM, while values >6 should lead to further investigation of peripheral limitations, such as endothelial dysfunction as a result of acute or chronic inflammation. In a sports cardiology setting, it is crucial to differentiate these findings from other transient conditions, such as overreaching syndrome or recent viral illness (including post-acute sequelae of COVID-19): As compared to suspected ACM, competitive athletes suffering from sequelae of COVID-19 may display ventilatory insufficiency, expressed by higher minute ventilation to carbon dioxide production (VE/VCO_2_), without exercise capacity limitations (23). In addition, athletes with overreaching syndrome may show more pronounced additional symptoms, such as fatigue, earlier increase of heart rate and a steeper incline of lactate concentrations (24) compared to ACM athletes. In addition to Q_c_/VO_2_, other CPET variables could be of use to differentiate cardiomyopathy from physiological adaptation, such as oxygen pulse and its kinetics, peak oxygen consumption and VE/VCO_2_ (25–29).

A diagnosis of definitive ACM also requires a proper analysis of arrhythmic burden at rest and during exercise by using Holter monitoring and CPET (Figure 1). Quantity, morphology, origin and increase of premature ventricular beats (PVB) during exercise must be considered in athletes. For instance, presence of PVBs with broad, right bundle branch block morphology are not to be expected in healthy athletes and may raise suspicion for left dominant ACM (especially when combined with low voltages or a ring-like pattern on magnetic resonance imaging) or non-ischemic cardiomyopathy (30–32). In ACM an increase of post exercise PVB conveys a worse prognosis in PKP-2 variant carriers (33). Thus, post exercise PVB should be rigidly analyzed in athletes not yet fulfilling diagnostic criteria for cardiomyopathy. An increase of polymorphic PVB during exercise should also raise suspicion of catecholaminergic polymorphic ventricular tachycardia (34). Due to the increasing knowledge of genetics in risk prediction and potential evolvement of a cardiomyopathy phenotype over time, repetitive clinical and genetic testing is warranted to unmask disease in athletes (11, 35–37). In summary, integrating several clinical aspects (potentially including Q_c_/VO_2)_ are necessary to elucidate the proper diagnosis during exercise testing.

Athletes not fulfilling diagnostic ACM criteria may cover a broad spectrum of clinical presentations. Q_c_/VO_2_ may be specifically helpful in clinical decision-making in the context of isolated right ventricular dilatation in the absence of clear T-wave inversions, or “atypical” left ventricular phenotypes. Criteria of (left and right) resting ejection fraction, which have been suggested in the task force and modified Padua criteria, can create a diagnostic dilemma in high-performance endurance athletes, as these may display lower-than-normal resting values. The diagnostic accuracy of these criteria may be improved by adding CPET echocardiography in athletes by showing preserved Q_c_/VO_2_ slope. This variable could, in addition to impaired right ventricular contractile function or strain during exercise, contribute to risk stratification in athletes with right ventricular arrhythmias (12, 18, 38, 39). Our proposed diagnostic algorithm to implement CPET echocardiography could provide deeper insights into exercise physiology of suspected cardiomyopathies by providing additional information on avDO_2_ and simultaneous analysis of potential mismatch between cardiac output and peripheral oxygen demand, which is not part of current sports cardiological routine assessment (4, 11).

### 3.3 Future research directions

Quantification of central limitations attributed to suspected ACM athletes may trigger closer follow-up exams. Further decline of Q_c_/VO_2_ upon repetitive exercise testing may make a diagnosis of ACM (or other cardiomyopathies) more likely and induce a more conservative approach to exercise recommendations in the athlete. As this hypothesis-generating assumption is not based on clinical data, but pathophysiological considerations, we suggest prospective assessment of athletes not fulfilling diagnostic criteria for ACM with combined CPET echocardiography to investigate its prognostic utility in terms of arrhythmogenic risk and development of heart failure. Whether Q_c_/VO_2_ slope has prognostic value in predicting ventricular arrhythmic events should be prospectively studied during long-term follow-up and compared to exercise (magnetic resonance) imaging. Studies should focus on investigating athletes with symptomatic and asymptomatic phenotype-positive, genotype-negative (including “exercise-induced cardiomyopathies”) as well as asymptomatic phenotype-negative, genotype-positive individuals. Exercise-induced response using Q_c_/VO_2_ may be an adjunct to repetitive morphological imaging and analysis of the arrhythmogenic burden during and after exercise to decide upon potential restriction from competitive sports, primary ICD-implantation, surveillance intervals and genetic cascade screening. In addition, the value of CPET may be enriched by including pulmonary lung function testing (40), or other important CPET variables, such as peak oxygen consumption, oxygen pulse and VE/VCO_2_ to differentiate physiological adaptation to exercise and the cardiomyopathic heart (28, 41).

### 3.4 Limitations of CPET echocardiography in suspected ACM

Despite being a cost and resource efficient way (compared to the costs of magnetic resonance imaging), combined semi-upright CPET echocardiography may be limited in resource-constrained settings. Although assessment of stroke volume should be feasible in athletes undergoing semi-upright CPET, calculation of biventricular ejection fraction, strain and other standard echocardiographic measurements may not always be possible. Obtaining reliable Doppler measurements of stroke volume at high intensity exercise and heart rates is challenging and may lead to misdiagnosis, warranting sufficient experience of the investigator. Performing such a study would require multicentre collaboration with expertise in combined CPET echocardiography. In addition, Q_c_/VO_2_ < 5 cannot be considered diagnostic of ACM as this can be present in other cardiomyopathies, inflammatory or myocardial storage diseases as well as sub-clinical left ventricular dysfunction. Although CPET echocardiography may be better feasible than exercise magnetic resonance imaging, this technique may only be available in highly specialized centers harbouring sufficient operator experience. However, in our experience and our countries, athletes under suspicion of cardiomyopathies are usually referred to such centers to provide the most accurate diagnosis, follow-up and (if necessary) treatment. Integration of clinical information, electrical findings, genetics, imaging and exercise data remains the domain of the clinical cardiologist. Clinical studies using standardized protocols are needed to validate the suggested approach. Q_c_/VO_2_ < 5 does not represent a clinically actionable threshold but a hypothesis generating variable requiring prospective validation.

### 3.5 Conclusions

In summary, evidence of how to deal with athletes under suspicion of ACM but not entirely fulfilling diagnostic criteria remains unclear and necessitates a better understanding of exercise pathophysiology and prospective trials. We suggest combined semi-upright CPET echocardiography to demonstrate mismatch between cardiac output and metabolic demand. Integrating CPET echocardiography in such cases may be feasible, help to better differentiate physiological adaptations from cardiomyopathic hearts, and may become a valuable adjunct for risk stratification and diagnostic frameworks.
